# Vitamin D status in mothers and their newborns in Iran

**DOI:** 10.1186/1471-2393-7-1

**Published:** 2007-02-12

**Authors:** Zhila Maghbooli, Arash Hossein-Nezhad, Ali Reza Shafaei, Farzaneh Karimi, Farzaneh Sadat Madani, Bagher Larijani

**Affiliations:** 1 Endocrinology and Metabolism Research Center, Tehran University of Medical Sciences, 5^th ^Floor, Shariati Hospital, North Kargar Avenue, Tehran 14114, Iran

## Abstract

**Background:**

Adequate vitamin D concentrations during pregnancy are necessary to neonatal calcium homeostasis, bone maturation and mineralization. The aim of study is to evaluate serum vitamin D concentrations in mothers and their newborns and effect of vitamin D deficiency on pregnancy outcomes.

**Methods:**

552 pregnant women were recruited from Tehran University educating hospitals in the winter of 2002. Maternal and cord blood samples were taken at delivery. The serum was assayed for 25-hydroxyvitamin D3, calcium, phosphorus and parathyroid hormone.

**Results:**

The prevalence of vitamin D deficiency in maternal and cord blood samples were 66.8% and 93.3%, respectively (<35 nmol/l). There was significant correlation between maternal and cord blood serum concentrations of vitamin D. In mothers with vitamin D deficiency, cord blood vitamin D concentrations was lower than those from normal mothers (P = .001). Also, a significant direct correlation was seen between maternal vitamin D intake and weight gain during pregnancy.

**Conclusion:**

Consideration to adequate calcium and vitamin D intake during pregnancy is essential. Furthermore, we think it is necessary to reconsider the recommendation for vitamin D supplementation for women during pregnancy.

## Background

Significant changes occur in maternal calcium metabolism during pregnancy. These changes occur to obtain the extra calcium needed for fetal skeletal growth [1,2]. Serum concentrations of 1,25(OH)2D increase 50–100% over the non-pregnant state during the second trimester, and by 100% during the third trimester [4,5]. The mechanism underlying the increased serum 1,25(OH)2D concentrations during pregnancy is not clear. PTH, which is usually considered the stimulus for increased renal hydroxylation of 25(OH)D to 1,25(OH)2D, has not been shown to be increased during pregnancy [4-6], and it has been speculated that the 1,25(OH)2D present in the maternal circulation may be of placental origin [7]. It is generally accepted that maternal vitamin D status during pregnancy reflects the maternal and neonatal calcium homeostasis [8-13].

Several studies have identified a surprisingly high prevalence of VDD in pregnant women in temperate regions such as the United Kingdom [11,14-17] and Norway [18], and even in sunny countries such as Pakistan [1], India [19], and Saudi Arabia [20]. In a pilot study in Iran, 80% of pregnant women had 25-hydroxyvitamin D [25(OH)D] concentrations less than 25 nmol/l. The mean cord serum 25(OH)D concentration was very low (4.9 +/- 9.4 nmol/l), and that from infants whose mothers had hypovitaminosis D were almost undetectable (1.2 +/- 1.2 nmol/l) [21]. Also, the prevalence of severe, moderate and mild vitamin D deficiency was 9.5%, 57.6% and 14.2%, respectively in a general Iranian population [22].

Several researches subsequently reported that infants of mothers with low vitamin D intake during pregnancy had low serum calcium concentrations in cord blood or during the first week of life [8,9,23]. Also, it may be possible that maternal vitamin D status affects fetal growth and bone development [24,25].

The aim of this study was to determine vitamin D status among pregnant women and their newborns. Also, we evaluated the effects of vitamin D status on pregnancy outcome.

## Methods

As a cross-sectional study, 567 pregnant women were recruited from Tehran University educating hospitals in the winter of 2002. After taking informal consent, 15 pregnant women were excluded from the study.

Blood samples were drawn and centrifuged for 30 minutes in the delivery room. Samples were frozen at -80 degrees centigrade in the Hormone Laboratory of the Endocrinology & Metabolism Research Center (EMRC).

The study protocol was approved by the research ethics committee of EMRC, the Medical Ethics Research Center and the ethics committee of the Iran Ministry of Health and Medical Education.

### Subjects

Subjects were healthy pregnant women and their newborns. Exclusion criteria were those with a known history or evidence of rheumatoid arthritis, thyroid, parathyroid or adrenal diseases, hepatic or renal failure, metabolic bone disease, type 1 diabetes mellitus, malabsorption, or medications influencing bone, vitamin D or calcium metabolism. Any kind of calcium or vitamin D supplementation in the current pregnancy was also noted.

### Measurement

Serum concentration of 25-hydroxy vitamin D_3 _was measured by a radioimmunoassay (RIA) method using a Biosource kit (Biosource Europe S.A, Belgium); intra- and inter-assay coefficients of variation (CV) were 5.2% and 7.5%, respectively (normal range: 2.5–75 ng/mL). Serum PTH was also detected using a Biosource kit (Biosource Europe S.A; normal range: 13–66 pg/ml), with intra- and inter-assay CV of 6.3% and 5.7%, respectively. Alkaline phosphatase was measured by enzymatic colorimetry using a Pars Azmoon kit (Pars Azmoon, Iran); Serum calcium and phosphorous were both measured by colorimetery using the Kavoshyar enzyme kit (Kavoshyar, Iran) and Sheem enzyme kit (Sheem enzyme, Iran), respectively.

Daily intake of dietary calcium and vitamin D was calculated from a food frequency questionnaire that was approved by the nutrition group of EMRC.

Newborn anthropometric measurements were performed at birth, with recording of head circumference to the nearest 1 mm (paper tape measure), length to the nearest 1 mm (Rollametre; Childhood Growth Foundation, London, United Kingdom), weight to the nearest 10 g (Seca, Birmingham, United Kingdom), and the largest diameter of anterior and posterior fontanels to the nearest 1 mm (paper tape measure).

Mothers' weight gains during pregnancy and infants' weights and gestational ages at birth were obtained from the medical records. LBW infants were classified as those whose weights were below 2500 g.

### Statistical analysis

We classified serum levels of 25(OH)D into four groups for deficiency status (<12.5 as severe, 12.5–24.9 as moderate, 25–34.9 as mild, and >34.9 nmol/L as normal). Data were analyzed using SPSS software, version 11.5. The student's T-test was used to compare the differences between the means of variables. The chi-square test was used to compare the frequency of variables. Pearson correlation was used to investigate correlation between two variables. In all tests, the level of significance was 0.05.

## Results

In total, 552 mothers participated in this study. Maternal and newborn characteristics are summarized in Table 1. There was no significant correlation between maternal serum vitamin D concentration with age (P = 0.2) or BMI (P = 0.2).

**Table 1:** Maternal and newborn characteristics

**Gestational age (week)**	38.38 ± 3.31*
**Parity**	1.02 ± 1.51*
**Gravity**	1.84 ± 1.91*
**Birth height (cm)**	50.02 ± 3.16*
**Birth weight (kg)**	3.19 ± 0.45*
**Preterm labor (<37)**	9.3%
**LBW (<2500)**	5.4%
**APGAR score**	8.7 ± 0.62*
**Cesarean section**	20.6%
**Adequate vitamin D intake**	50.1%
**Adequate calcium intake**	55.9%

* Mean ± 2SD

The mean maternal serum 25(OH)D concentration was 27.8 ± 21.71 nmol/l, and the prevalence of vitamin D deficiency in mothers was 66.8% (<35nmol/l), while only 3.4% of pregnancies had serum levels of 25(OH)D higher than 80 nmol/l. Of note, the prevalence of severe, moderate, and mild vitamin D deficiency in newborns was 33.8%, 51.8%, and 7.7%, respectively.

Levels of maternal and cord blood biochemical markers are summarized in Table 2. Serum vitamin D concentrations and ALP were inversely correlated with each other in mothers (r = -.265, p = .001), and there was also an inverse correlation between serum vitamin D concentrations and PTH in mothers (r = -.18, P = .002). No correlation was seen between serum vitamin D concentrations and calcium or phosphorus.

**Table 2:** Levels of maternal and cord blood biochemical markers

	**Maternal**	**Newborn**
**25 (OH) D (nmol/l)**	27.82 ± 21.71*	18.1 ± 11.6*
**Ca (mmol/l)**	9.03 ± 1.3*	10.58 ± 1.38*
**ALP(IU/l)**	539.47 ± 274.32*	520.78 ± 347.6*
**PTH (pg/ml)**	23 ± 19.7*	16 ± 14.34*

* Mean ± 2SD

43.5 percent of women had received adequate vitamin D and calcium intake during pregnancy. No significant correlation was observed between maternal and cord blood serum vitamin D levels with calcium and vitamin D intake (P = 0.1). Accordingly, maternal serum vitamin D strongly correlated with cord blood vitamin D levels (p = 0.0001, r = 0.706). There was also a significant correlation between maternal and cord blood calcium levels (r = 0.23, P = 0.002).

Significant inverse correlations were seen between cord blood levels of vitamin D and ALP (r = -0.29, p = 0.0001) and PTH (r = -0.158, p = 0.03). In addition, a significant correlation was observed between cord blood calcium concentrations and ALP (P = .04, r = .145), and also between PTH and ALP in cord blood (P = 0.006, r = 0.2). There was no significant correlation between maternal and cord blood PTH concentrations (P = 0.1).

Among the newborns of mothers who had vitamin D deficiency, vitamin D concentrations in cord blood were lower than newborns of normal mothers (15.6 ± 7.2 vs. 20.2 ± 14.9, p = .009). Among the newborns with severe vitamin D deficiency (<12.5 ng/ml), serum ALP was higher (331.4 ± 95 compared with 606.9 ± 270.3, p = 0.001), while there was not a significant different in PTH or calcium in cord blood.

### Anthropometric measurements

A significant direct correlation was seen between maternal vitamin D intake and weight gain during pregnancy (r = .11, p = .01), and also between maternal ALP and birth weight (r = .014, p = .02). But there was no significant effect of maternal vitamin D deficiency on weight gain during pregnancy, and there was no correlation between maternal calcium and weight gain.

No significant correlation was observed between maternal and cord blood vitamin D concentration and the newborns' characteristics (weight, height, head circumference, posterior and anterior fontanel diameter and APGAR). Conversely, maternal PTH directly correlated with posterior (r = .21, p= .001) and anterior (r = .18, p = .005) fontanel diameter. Furthermore, no significant correlation was seen between cord blood calcium and newborns' characteristics. In contrast, the diameter of the posterior fontanel was significantly wider in newborns of mothers with vitamin D deficiency than those of normal mothers. Finally, no significant correlation was observed between LBW and cord blood or maternal vitamin D deficiency.

## Discussion

Our findings reveal a high prevalence of vitamin D deficiency among pregnant women and newborns. Almost two in three mothers had vitamin D deficiency (VDD), while one in ten newborns had normal vitamin D. Vitamin D deficiency in pregnant women and newborns has been reported in several studies [18,26-28]. This prevalence is reported based on an old definition of vitamin D deficiency, and many investigators now define deficiency as <80 nM (32 ng/mL) circulating 25(OH)D/L [29,30]. Based on this cut-off, only 4% of pregnancies in our study had normal serum vitamin D concentrations. It seems most studies define the cut-off point for vitamin D and calcium intake based on serum vitamin D and calcium concentrations. Thus new cut off point definition based on outcomes may be more suitable [12,23,31-34]

Our results add to the evidence that cord blood 25(OH)D strongly correlated with maternal values, as reported by several studies [15,19,23,26,27,35,36]. In this study we found a significant negative correlation between maternal vitamin D and PTH concentrations, in agreement with the other studies [19,35-37]. Studies have shown that long-term vitamin D deficiency can result in increased PTH concentrations and decreased serum 1,25(OH)2D concentrations, which leads to osteomalacia [38]. In contrast, Datta et al. recently screened 160 pregnant women from ethnic minority groups in South Wales and found that 50% had low serum 25(OH)D concentrations, whereas PTH concentrations were within the normal range for 81% of the women with low 25(OH)D concentrations [11].

In the current study there was a weak inverse correlation between maternal serum ALP and vitamin D concentrations, which confirms the results of other studies. Brooke et al. [15] reported elevation of ALP in 20% of Asian subjects from the United Kingdom with serum 25(OH)D concentrations below 25 nmol/L. Rab and Baseer [42] from Pakistan reported elevated total ALP in 26% of pregnant women with low daily vitamin D intake, but serum 25(OH)D was not measured. Marya et al. [9] from India reported elevated ALP in 13% of their pregnant subjects who were not receiving vitamin D supplementation, whereas none of the subjects supplemented with vitamin D had elevated ALP.

Our results indicated that newborns of mothers with an adequate intake of vitamin D had higher cord blood vitamin D. Similarly, Delvin et al. assessed the effects of supplementation with 1000 IU/d vitamin D compared to placebo; in the treatment group, the cord blood vitamin D concentration was 45 nmol/l, three times which of the control group [39].

In this study, among the newborns with severe vitamin D deficiency, serum PTH was found to be significantly high, although there was no significant correlation between serum vitamin D levels and PTH in cord blood, consistent with other of studies [40-42]. Furthermore, Okonofual et al. performed a study comparing Asian (with inadequate vitamin D intake) and white (with adequate vitamin D intake) mothers, and found lower serum 25(OH)D concentrations and higher PTH concentrations in cord blood samples from Asian neonates compared with white neonates [10].

In our study, there was a significant correlation between the newborns' vitamin D and calcium concentrations. Some studies have shown that maternal vitamin D status may influence placental calcium transfer [10,43]. Namgung et al, in a study of Korean newborn infants born in the winter, found that 97% had markedly lower serum 25(OH)D concentrations. This may reflect decreased synthesis in the fetal kidney associated with limited 25(OH)D substrate availability, and theoretically could be a factor affecting placental calcium transfer and fetal bone mineralization [42]. Consequently, most studies report that maternal vitamin D concentration plays a crucial role in neonatal and maternal calcium homeostasis, and that infants of mothers with low vitamin D intake during pregnancy had low serum calcium concentrations in cord blood or during the first week of life [8,9,23].

In our study, there was no significant correlation between maternal calcium or vitamin D with maternal weight gain during pregnancy or birth weight. Also, we didn't find any significant correlation between LBW with cord blood and maternal vitamin D deficiency. A few studies showed that maternal vitamin D deficiency might affect maternal weight gain or fetal growth. Two studies by Marya et al. [9,13] revealed a beneficial effect of vitamin D supply on birth weight, whereas others did not [28,29,33]. Also, Marya et al. [13] found no effect of maternal vitamin D supplementation on weight gain, while Brooke et al. observed less weight gain in the pregnancies without supplementation compared with the supplementation group [44].

Our findings showed that the diameter of fontanel was significantly wider in newborns of mothers with vitamin D deficiency than newborns of normal mothers. In follow-up assessments by Brook et al., neonates in the placebo group also had a greater fontanel area than did the supplemented group [15]. Also, in follow-up assessments by Maxwell et al., an increase in the size of the fontanel was noted among the infants of the control mothers vs. those of the treatment mothers [45]. Another study of 256 term infants conducted in China also found possible evidence for a relationship between maternal vitamin D deficiency and impaired fetal bone ossification [46]. Although Brooke et al. did not find an association between craniotabes and vitamin D status; they concluded that infants of mothers who received placebo had a larger fontanel than infants of mothers were treated with vitamin D, which is consistent with impaired ossification of the skull. In a recent study, there was a negative correlation between maternal vitamin D concentrations and the largest diameter of the inferior fontanel area [15].

In our study, there was no significant effect of maternal vitamin D deficiency on weight gain during pregnancy. Furthermore, there was no significant correlation between maternal vitamin D deficiency with the newborn's weight, height, or head circumference. In follow-up assessments by Shefras et al., there was a significant association between vitamin D received and head circumference, which remained after adjustment for confounding factors [47]. Also, Marya et al. reported infants of mothers who received vitamin D had a greater head circumference compared with infants of mothers who did not receive vitamin D [13]. The cut-off point for vitamin D deficiency may have an influence on detectable changes in pregnancy outcomes.

## Conclusion

We conclude that vitamin D deficiency is frequent in mothers and newborns, and correlates with inadequate vitamin D and calcium intake. Because of this, vitamin D supplements have an important role in pregnant women in decreasing the risk of subsequent complications.

## Competing interests

The author(s) declare that they have no competing interests.

## Authors' contributions

ZM carried out the designing of protocol, participation in data collection, and analysis

AH carried out the designing of protocol, participation in data collection, and analysis

ARS carried out the data collection and laboratory tests

FK carried out the laboratory tests

FSM participation in data collection

## Pre-publication history

The pre-publication history for this paper can be accessed here:


